# Pesci: fast and user-friendly software to compare single-cell gene expression across species

**DOI:** 10.1093/bioinformatics/btag623

**Published:** 2026-08-19

**Authors:** Elise Parey, Laura Piovani, Ferdinand Marlétaz

**Affiliations:** Centre for Life’s Origins and Evolution (CLOE), Department of Genetics, Evolution & Environment, University College London, London, WC1E 6BT, United Kingdom; Centre for Life’s Origins and Evolution (CLOE), Department of Genetics, Evolution & Environment, University College London, London, WC1E 6BT, United Kingdom; Centre for Life’s Origins and Evolution (CLOE), Department of Genetics, Evolution & Environment, University College London, London, WC1E 6BT, United Kingdom

## Abstract

**Summary:**

Recent technological advances have propelled comparative functional genomics into the single-cell era, spurring a rapid development of methods to analyse these complex datasets. However, comparing single-cell gene expression across species to quantify expression similarity and ultimately identify homologous cell types remains an open problem. The ICC algorithm (Iterative Correlation of Coexpression) has been recently proposed as an attractive approach to tackle this challenge, but, to date, no software implementation is available. Here, we introduce Pesci (Pretty Easy Single-cell Comparisons using ICC), an efficient and user-friendly implementation of the ICC algorithm applied to pairwise comparisons of single-cell gene expression atlases across species.

**Availability:**

Pesci is implemented in Python 3 (≥3.7). It is available for download on Linux, macOS and Windows via pip, conda and GitHub at https://github.com/eparey/pesci. The source code is permanently archived on Zenodo (https://doi.org/10.5281/zenodo.21477543).

## 1 Introduction

The advent of single-cell transcriptomics has enabled the construction of cell atlases for animals across the Tree of Life (Tabula Muris Consortium *et al.* 2018, Musser *et al.* 2021, Li *et al.* 2022, Najle *et al.* 2023, Sebé-Pedrós *et al.* 2025), offering unprecedented opportunities to decipher the rules that govern the evolution of cell types and the emergence of complex organs. However, comparing single-cell gene expression across species remains a significant challenge, especially at large evolutionary distances. These difficulties are due, in part, to the inherent sparsity of single-cell data and the divergence of gene sequence and gene expression over time that can obscure common ancestry. This is particularly challenging in distantly-related species, which typically share fewer 1-to-1 orthologous genes than closely related ones, limiting the number of genes that can be included in comparisons.

Recently, (Najle *et al.* 2023) employed a new approach to compute gene expression similarity across single-cell datasets of different species and trace shared evolutionary ancestry of cell types through expression conservation. In order to leverage both 1-to-1 and many-to-many orthologous genes in cross-species single-cell comparisons, (Najle *et al.* 2023) adapted the ICC procedure (Iterative Correlation of Co-expression) first proposed by (Tirosh and Barkai 2007). Single-cell ICC represents an alternative to SAMap (Tarashansky *et al.* 2021), the most widely-used tool to compare single-cell gene expression atlases across species.

Single-cell ICC and SAMap differ in two main aspects. Firstly, SAMap is an integration-based method (embeds expression profiles of cells of the two species in one joint space), whereas ICC is used in an aggregation-based framework (gene expression profiles are aggregated across cells of the same metacell or cell cluster before comparisons across species). Secondly, SAMap and single-cell ICC use different approaches to incorporate additional homologous genes that are not 1-to-1 orthologs. SAMap considers all genes identified by significant blast hits, which can include many-to-many orthologs as well as more distant out-paralogs (Gabaldón and Koonin 2013). ICC doesn’t consider out-paralogs and instead picks only the “best” (most similar in expression) gene pair from many-to-many orthologs groups. The underlying rationale is that, within many-to-many orthologs, the pair with the most similar expression most likely reflects the ancestral gene expression profile. Tracking this single ancestral gene copy as a unique signal makes ICC less prone to cell cluster matches driven by independent species-specific duplications and convergent evolution (Stadtmauer *et al.* 2025).

Unlike SAMap, there is currently no software implementation for single-cell ICC. In the context of fast-paced single cell data generation, efficient and easy-to-use software is crucial to fuel biological discoveries. Moreover, alternative approaches for comparing cell expression between species are essential for more accurate interpretation of data across biological contexts. To this end, we introduce Pesci (Pretty Easy Single-cell Comparisons using ICC), a fast and user-friendly implementation of the ICC algorithm for single-cell gene expression comparison.

## 2 Implementation

Pesci takes as input gene-by-cell expression count matrices (with or without replicates) and pre-computed cell clusters for the two species to compare, along with gene orthologies. Pesci is flexible in its input formats, accepting count matrices provided in any of the three most frequently used formats, that is, either: (i) a raw dense count matrix in text format—commonly found in GEO (Barrett *et al.* 2013), (ii) a CellRanger directory (Zheng *et al.* 2017) or (iii) a Scanpy h5ad file (Wolf *et al.* 2018). We provide instructions in Pesci’s documentation to easily convert a Seurat object (Hao *et al*. 2024) into one of the accepted formats. Gene orthologies and cluster annotations can be provided as simple text files.

We implemented Pesci following the procedure described in (Najle *et al.* 2023). Briefly, the workflow consists of three main steps: (1) cell aggregation to obtain a normalised gene-by-cluster expression matrix for each species, (2) selection of orthologs to use for comparisons (including many-to-many orthologs) and (3) cross-species cell cluster gene expression comparison.

### 2.1 Gene-by-cluster expression matrix

Pesci first computes gene-by-cluster expression from the gene-by-cell UMI count matrix, as the geometric mean of UMI count values over cells of the same cluster. In a second step, the matrix is normalised for each gene by its median of expression across clusters. The resulting matrix captures gene expression fold-change, where higher values are assigned to marker genes in the cluster they are specific to. This ensures that marker genes contribute more to expression similarity scores that will be computed in step 3.

### 2.2 Ortholog selection

Below is a brief overview of how the ICC procedure is able to select the pair of genes with highest expression similarity among many-to-many orthologs, without having to first match single-cell clusters across species. This is achieved by computing an Expression Conservation (EC) score, also called co-correlation score, for each possible pair of orthologs from the many-to-many group, and then retaining the pair with the highest score.

For the sake of simplicity we describe here the EC score using the example case of one-to-one orthologs. First, for each species, a Pearson correlation matrix is computed from the cell-by-cluster matrix (or a cell-by-metacell matrix, if provided), using one-to-one orthologs only. For instance, for a dataset with *n* = 2000 one-to-one orthologs, such a matrix for species 1 would have dimension 2000 × 2000, where the value in the cell (*i*, *j*) is the expression correlation between genes *i* and *j*. Second, the two correlation matrices (one per species) are sorted to store orthologs at the same indexes and compared row-wise with Pearson correlation again (correlation of row i from species 1 and row i for species 2, where i correspond to the index of ortholog gene *i*). This results in an EC score for each one-to-one ortholog (i.e. a vector of *n* = 2000 EC scores in the example above). The EC score measures expression similarity for a pair of orthologous genes, as given by their co-correlation. Step 2 is repeated using weighted Pearson correlation with weights for each orthologous gene pair given by their EC score of the previous iteration, until convergence is reached.

The procedure described above is then adapted to the case of many-to-many orthologs by padding the input matrix to accommodate all possible 1–1 gene pairings. An EC score is computed as described above for each possible pairing, and the pair with highest EC is retained for cross species comparisons.

## 3 Optimisation and flexibility

Pesci is implemented in Python 3 (≥3.7) and uses Einstein summation notations available in numpy for efficient matrix operations (Einstein 1916, Harris *et al.* 2020), allowing it to run in just a few minutes on a laptop (see Applications section). Pesci is easy-to-use and flexible, accommodating a wide range of input formats and coming with detailed documentation and many tunable parameters. For instance, command-line arguments are available to customise heatmap figure generation (output formats, heatmap ordering), to compare only a subset of the cell clusters, to use one-to-one orthologs only or to tune marker specificity.

## 4 Applications

We validated Pesci and tested its performance on four real datasets: placozoa scRNA-seq from (Najle *et al.* 2023), lophotrochozoan larvae scRNA-seq from (Piovani *et al.* 2023), mammalian retinal bipolar cell subtypes from (Hahn *et al.* 2023) and fruit fly cell types from the Fly Cell Atlas (Li *et al.* 2022). Except where stated otherwise, all applications presented below were run on 4 cores (—cores 4) on an Apple M2 Pro laptop with 32GB of RAM and a 10 cores CPU, and using the least-optimal input data format (dense cell-by-gene expression matrices in text format).

First, to validate the correct implementation of the ICC algorithm in Pesci, we applied it to the comparison of 4 placozoa whole-body single-cell expression atlases (∼ 15k cells per species) from (Najle *et al.* 2023). As expected, Pesci correctly reproduced results from (Najle *et al.* 2023) (Fig. S1, available as supplementary data at *Bioinformatics* online), running in only 2 minutes to perform all six pairwise comparisons (total elapsed time: 125.08 s, total CPU time: 375.55 s).

Second, we tested Pesci on data from (Piovani *et al.* 2023), comparing whole body atlases for an oyster larva (8.5k cells) and a flatworm larva (17.6k cells). On these datasets, Pesci ran in less than 1 minute (total elapsed time: 48.71 s, total CPU time: 154.52 s). To evaluate efficiency on a larger dataset, we artificially up-sampled this data to reach 200k cells per species. Pesci ran in less than 5 minutes (total elapsed time: 271.93 s, total CPU time: 617.47 s).

**Figure 1:**
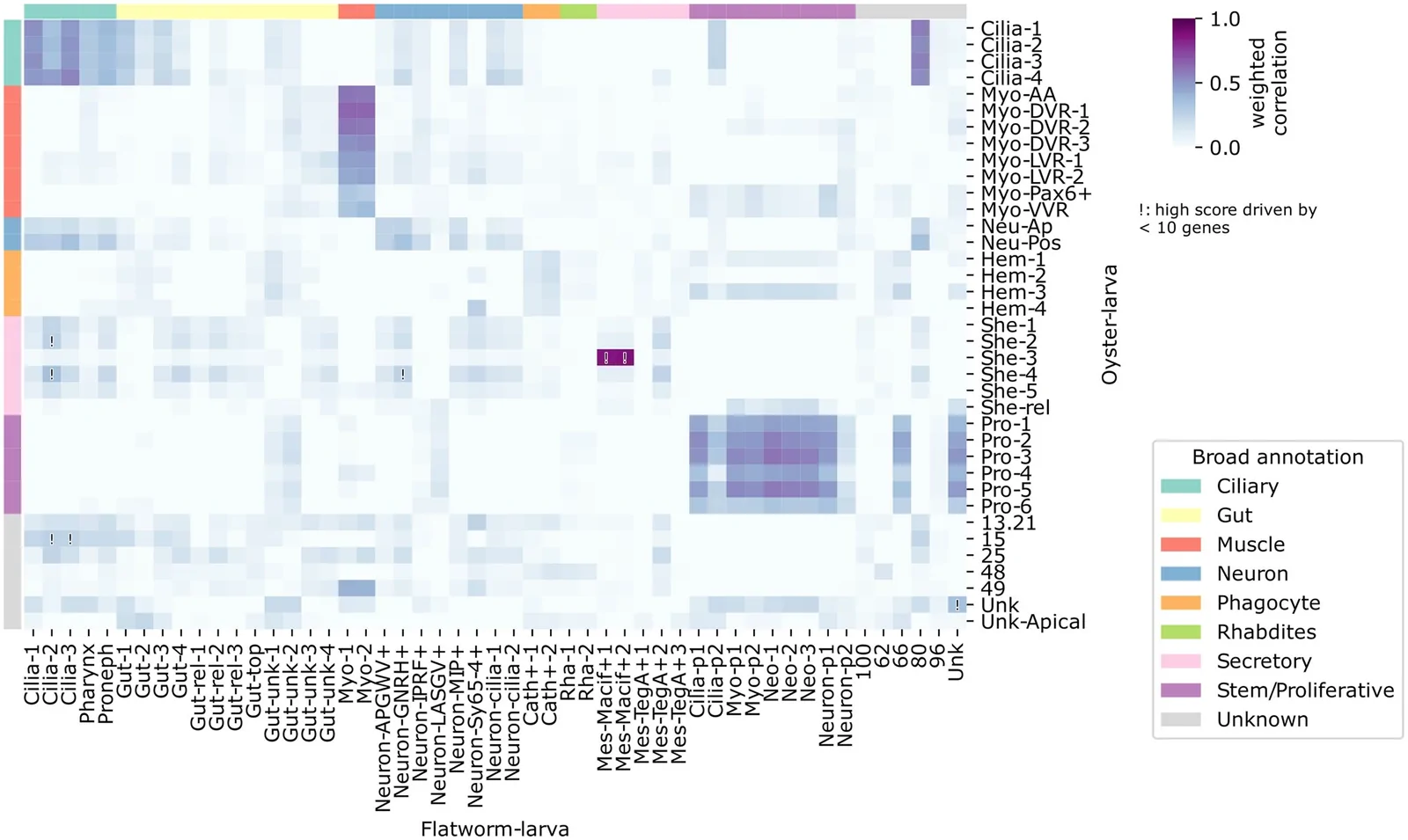
Example of Pesci output, comparing gene expression across cell clusters of oyster and flatworm larvae. Pesci was run using the original raw count data from (Piovani *et al*. 2023). Cell clusters of the two datasets are ordered by user-supplied broad cell type annotations. Automatic warnings, displayed as exclamation marks, are shown where a high score is driven by a small number of co-expressed orthologs (<10), as is the case for Flatworm Mes-Macif + cells and Oyster She-3 cells in this example. Warnings warrant careful interpretations of cell matches and inspection of the co-expressed marker genes (Table S1, available as supplementary data at *Bioinformatics* online, Figure S2f).

Overall, results matched those reported using SAMap (Piovani *et al.* 2023). We recovered high gene expression similarity across cilia, myocytes, secretory and proliferative cells in the two species, suggesting their homology (Fig. 1). Moreover, most neuronal and phagocytic cell clusters of the oyster larva respectively exhibited maximal reciprocal similarity with neurons and phagocytes of the flatworm larva, but expression similarity was weaker than in the aforementioned cell types.

We next compared the co-expressed marker genes reported by Pesci and SAMap. To facilitate biological interpretation of the reported cell cluster matches, both Pesci and SAMap report gene pairs that are driving the cell cluster match scores (positive co-correlation). However, SAMap restricts this list to only report genes that are also differentially expressed (DE) in corresponding cell clusters of each species. Because this can obscure genes contributing to cell cluster correlation scores, in Pesci, by default, we more comprehensively report co-expressed gene pairs displaying an expression fold-change fc > 1.5. These can then be further filtered using DE tests or more stringent thresholds. In the oyster-flatworm comparison, Pesci reported a higher number of co-expressed marker gene pairs despite considering substantially less gene pairs (*n *= 223 424 in the refined SAMap graph vs *n* = 6222 in Pesci), supporting that Pesci does not miss key conserved markers (Tables S1 and S2, Fig. S2a–c, available as supplementary data at *Bioinformatics* online). Crucially, Pesci co-expressed markers were also significantly more specific than SAMap markers (Fig. S2c, available as supplementary data at *Bioinformatics* online). Lastly, on this data, and despite Pesci not performing DE tests, the vast majority of Pesci markers were also statistically supported to be differentially expressed (96%, Fig. S2d, available as supplementary data at *Bioinformatics* online).

Altogether, Pesci reports similar gene expression conservation as SAMap, while offering more control on the orthologous genes selected and reporting more and higher specificity conserved markers.

Third, to illustrate Pesci’s suitability to matching cell subtypes at closer evolutionary distances, we applied it to the mammalian retina single-cell expression atlases from (Hahn *et al.* 2023). Specifically, we compared bipolar cell subtypes identified in the Cow retina (92k total cells, 23k bipolar cells) with subtypes identified in the Ferret retina (64k total cells, 8k bipolar cells). On these datasets, Pesci ran in approximately 2 minutes (total elapsed time: 142.58 s, total CPU time: 320.40 s). Pesci successfully matched the orthologous subtypes that were previously identified by (Hahn *et al.* 2023) who used multispecies integration of bipolar cells sampled across 17 vertebrate species (Figs S3A-B, S4, available as supplementary data at *Bioinformatics* online). In cross-mammal comparisons like this one, Pesci could also be used in combination with integration-based approaches to increase the number of genes used. For instance, Pesci’s ortholog selection step could be run first, and its output used for feature selection and integration.

Fourth, while not its primary use case, Pesci can be applied to compare single-cell expression across different organs of a single species. Here, gene expression similarity is computed using Pearson weighted correlation as previously described, but genes are directly matched by name across single-cell datasets (i.e. bypassing ortholog-related steps). To test this, we applied Pesci to the comparison of adult fruit fly organs datasets from the Fly Cell Atlas (Li *et al.* 2022), comparing adult fly trachea with adult fly proboscis (26k cells each). This comparison ran in approximately 30 seconds using the optimal sparse matrix .h5ad input format (total elapsed time: 17.38 s, total CPU time: 20.64 s) and Pesci successfully matched identical cell types across compared organs (Fig. S3C, available as supplementary data at *Bioinformatics* online).

Finally, for newly generated datasets, Pesci provides a convenient first-pass framework for broad cell annotation through comparison with reference annotated atlases from other species. These provisional annotations can then be refined through inspection of the conserved marker gene lists provided.

## 5 Conclusion

Here we present Pesci, a user-friendly and efficient software tool to compare single-cell gene expression across pairs of species. As demonstrated, Pesci runs in less than a minute on a laptop and supports input cell-by-gene expression matrices in the most-frequently used formats. We show that Pesci can be used on different biological datasets (whole body and tissue-level atlases) and across a wide range of evolutionary distances (from placental mammals diverged ∼80 MYA to spiralians diverged ∼500 MYA). Its ease of use and versatility make single-cell gene expression comparison accessible to a broader user base, which is essential for driving biological discoveries. In addition, Pesci provides an alternative aggregation-based approach to cross-species single-cell comparisons, expanding the toolbox for single-cell expression analysis and facilitating future methodological developments in the field.

## Author contributions

Elise Parey (Conceptualization [Equal], Formal analysis [Equal], Funding acquisition [Equal], Methodology [Equal], Software [Equal], Writing—original draft [Equal]), Laura Piovani (Conceptualization [Equal], Formal analysis [Equal], Methodology [Equal], Writing—review & editing [Equal]), and Ferdinand Marlétaz (Conceptualization [Equal], Funding acquisition [Equal], Writing—review & editing [Equal])

## Supplementary Material

btag623_Supplementary_Data

## Data Availability

No new data were generated in support of this work. Pesci was evaluated using the published datasets available in the referenced studies.
