# Supplementary material for: Pesci: fast and user-friendly software to compare single-cell gene expression across species
Source: Bioinformatics. 2026 Aug 19;42(9):btag623. doi: 10.1093/bioinformatics/btag623 (PMC13537516; doi:10.1093/bioinformatics/btag623)
Supplement: Supplementary file 1 [file btag623_supplementary_data.pdf]

SUPPLEMENTARY MATERIAL

Pesci: fast and user-friendly software to compare single-cell gene expression across species

Parey et al.

List of Supplementary Tables

Table S1: Conserved marker genes identified by Pesci and SAMap in the Flatworm-Oyster comparison

Table S2: Number of orthologous gene pairs used by Pesci in the different cross-species comparisons

List of Supplementary Figures

Figure S1: Comparison of gene expression across cell clusters of 4 placozoan species with Pesci

Figure S2: Conserved marker genes in the Flatworm-Oyster comparison identified by Pesci and SAMap for the cilia cell clusters

Figure S3: Additional use cases of Pesci: comparison of mammalian cell subtypes and comparison of fruit fly organs

Figure S4: Hierarchical clustering of cell expression profiles for the placozoa whole-body atlas and the mammalian retina dataset

Cell type	# Pesci and SAMap markers	# Pesci only markers	# SAMap only markers
Cilia	60	126	77
Neuron	15	104	36
Myocyte	50	159	111
Stem	134	329	56
She3/Macif	3	4	5

Table S1: Conserved marker genes identified by Pesci and SAMap in the Flatworm-Oyster comparison.

Dataset	Source	Species 1	Species 2	# orthologous gene pairs
Lophotrochozoa larvae (whole-body)	(Piovani *et al.*, 2023)	Pacific oyster (*Magallana gigas*)	Tiger flatworm (*Prostheceraeus crozieri*)	6,222
Placozoa (whole-body)	(Najle *et al.*, 2023)	*Trichoplax* H1	*Trichoplax* H2	8,402
Placozoa (whole-body)	(Najle *et al.*, 2023)	*Trichoplax* H1	*Hoilungia* H13	7,221
Placozoa (whole-body)	(Najle *et al.*, 2023)	*Trichoplax* H1	*Cladtertia* H23	7,659
Placozoa (whole-body)	(Najle *et al.*, 2023)	*Trichoplax* H2	*Hoilungia* H13	9,049
Placozoa (whole-body)	(Najle *et al.*, 2023)	*Trichoplax* H2	*Cladtertia* H23	8,535
Placozoa (whole-body)	(Najle *et al.*, 2023)	*Hoilungia* H13	*Cladtertia* H23	8,288
Mammals (retinal BP cells)	(Hahn *et al.*, 2023)	Cow (*Bos taurus*)	Ferret (*Mustela putorius furo*)	12,751

Table S2: Number of orthologous gene pairs used by Pesci in the different cross-species comparisons.

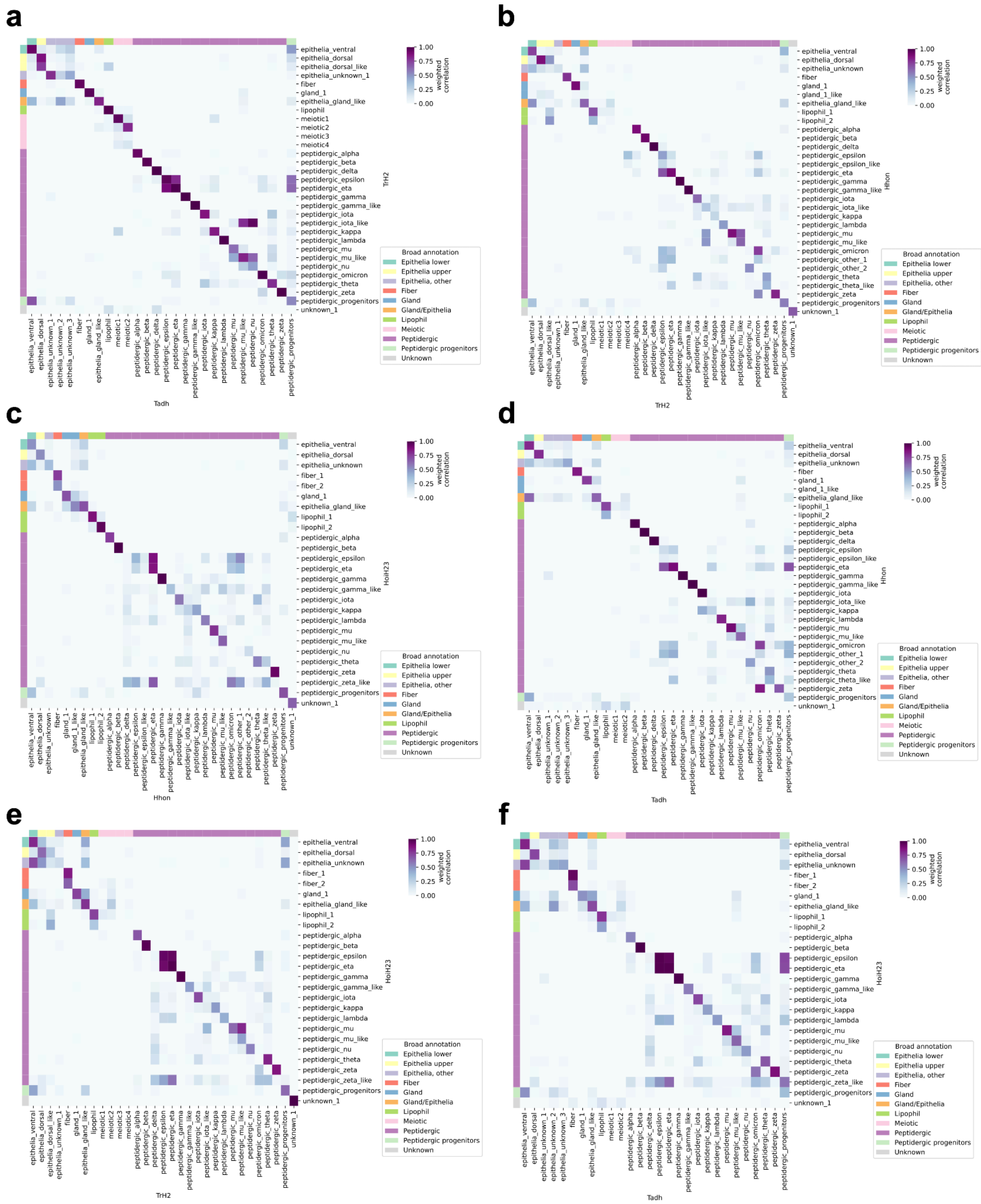

<u>Figure S1:</u> Comparison of gene expression across cell clusters of 4 placozoan species with Pesci. Single-cell expression data from (Najle *et al.*, 2023). **a.** *Trichoplax* H1 (Tadh) vs *Trichoplax* H2 (TrH2). **b.** *Trichoplax* H2 (TrH2) vs *Hoilungia* H13 (Hhon). **c.** *Hoilungia* H13 (Hhon) vs *Cladtertia* H23 (HoiH23). **d.** *Trichoplax* H1 (Tadh) vs *Hoilungia* H13 (Hhon) **e.** *Trichoplax* H2 (TrH2) vs *Cladtertia* H23 (HoiH23). **f.** *Trichoplax* H1 (Tadh) vs *Cladtertia* H23 (HoiH23).

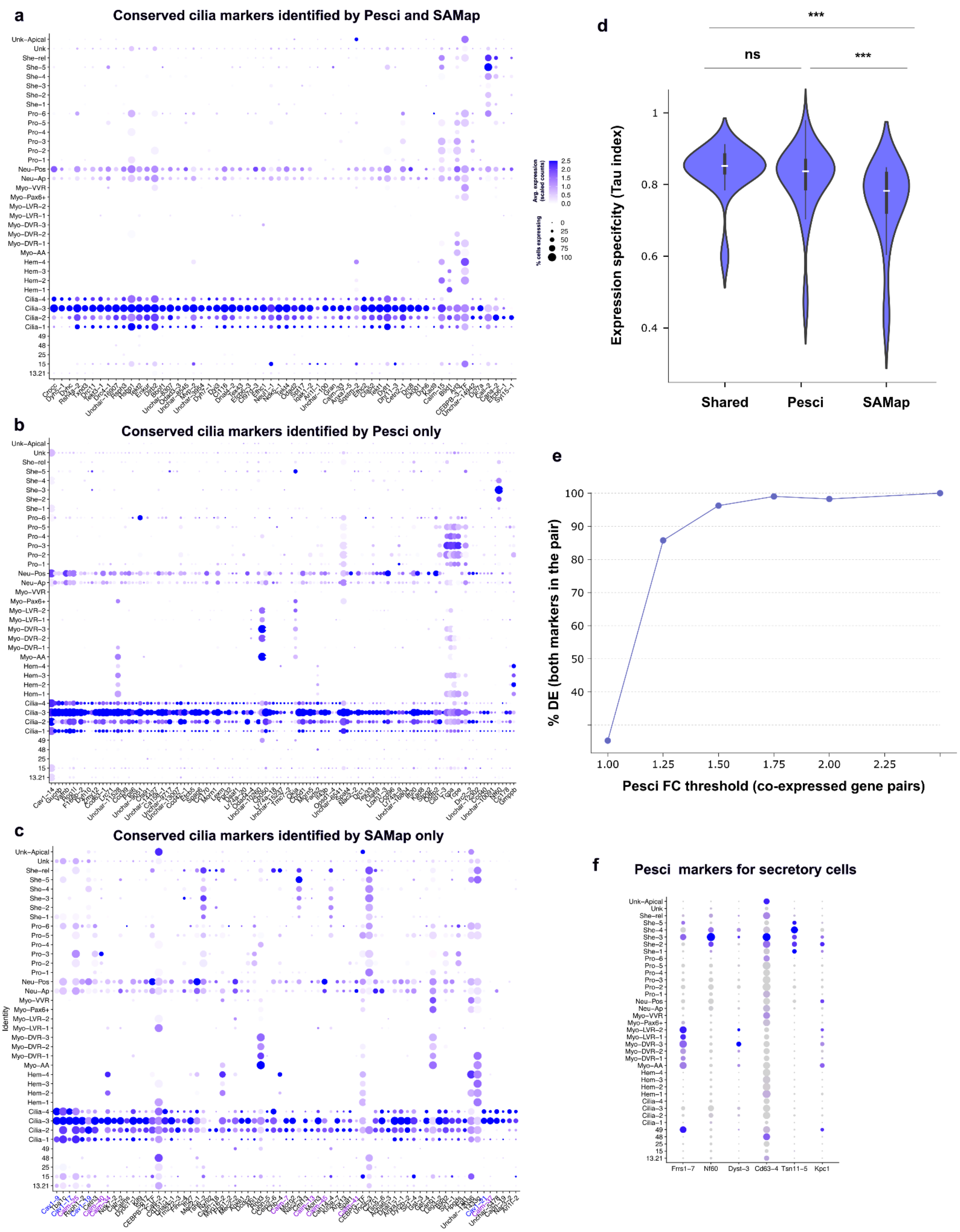

Figure S2: Conserved marker genes in the Flatworm-Oyster comparison identified by Pesci and SAMap for the cilia cell clusters. **a.** Dotplot of gene expression (z-score of average log-normalised counts, clipped between 0 and 2.5) in the Oyster, for the conserved cilia markers identified by both Pesci and SAMap (n=60) (Tarashansky *et al.*, 2021). Pesci and SAMap were both run with default parameters for conserved marker identification. **b.** As in **a** but for conserved markers identified by Pesci only (n=126). **c.** As in **a** but for conserved markers identified by SAMap only (n=77). SAMap includes duplicates of the same gene families. For instance, Cav1 and Calm numerous copies (4 and 8 copies, respectively) are

shown in dark blue and purple respectively. **d.** Tau specificity index for co-expressed markers identified by both methods (Shared) or only by Pesci or SAMap. Higher tau values indicate higher cell-specific expression. Pesci-only markers are significantly more specific than SAMap-only markers (two-tailed Mann-Whitney U test, *$p < 0.05$, **$p < 0.01$, ***$p < 0.001$, ns = not significant). **e.** Percentage of all co-expressed cilia marker gene pairs reported by Pesci (n=186) in the oyster-flatworm larva comparison that are also differentially expressed in corresponding cilia clusters (Wilcoxon rank sum tests as implemented in Seurat, adjusted p-value $p < 0.05$). On this dataset, 96% of Pesci marker pairs reported at the default 1.5 fold-change threshold are also differentially expressed. Based on DE tests performed by (Piovani et al. 2023) with stricter parameters (p-val < 10^{-2} and %cells expressing > 25), 73% of all Pesci markers (default threshold) are also defined as differentially expressed in cilia clusters in both species. **f.** Dotplot of gene expression in the Oyster, for the few markers driving the low-confidence She-3/Mes-Macif+2 match (highlighted by a warning in Pesci output, see **Figure 1**).

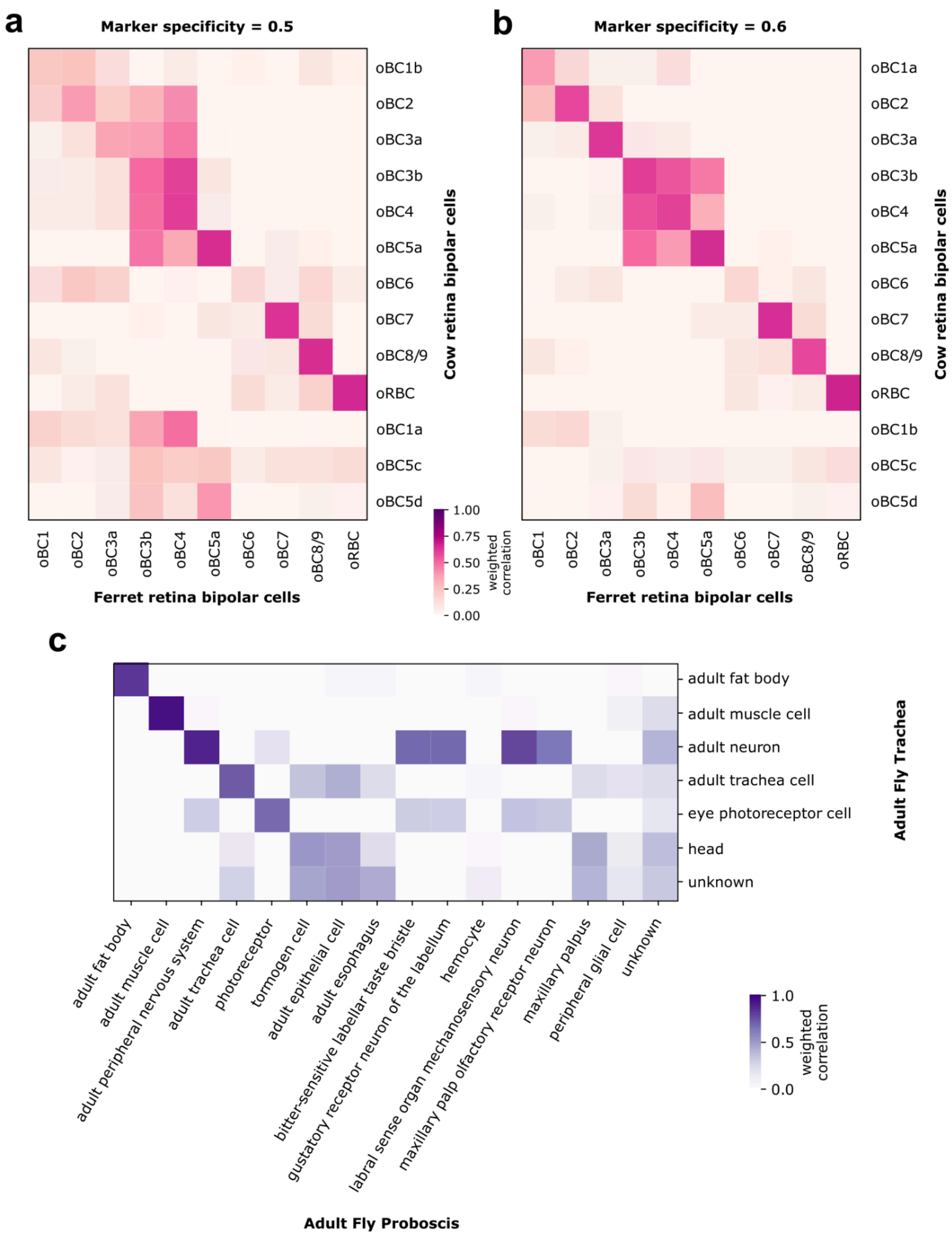

Figure S3: Additional use cases of Pesci: comparison of mammalian cell subtypes and comparison of fruit fly organs. **a.** Comparison of retinal bipolar cell subtypes between cow and ferret. Single-cell expression data from (Hahn *et al.*, 2023). The heatmap is automatically ordered by Pesci to display best 1-1 matches on the diagonal and correspond to the orthologous subtypes annotated by (Hahn *et al.*, 2023) (Cow oBC1a, oBC5c and OBC5d are not found in the Ferret retina and are therefore not part of the main diagonal). **b.** Comparison of retinal bipolar cell subtypes between cow and ferret, as in **a** but increasing Pesci's marker specificity parameter. Increasing contribution of higher specificity markers improves similarity scores for the 1-1 matches shown on the diagonal. **c.** Comparison of cellular gene expression in the trachea and proboscis of the adult fruit fly (Li *et al.*, 2022). Similarly as in **a** and **b**, best 1-1 matches are automatically displayed on the diagonal.

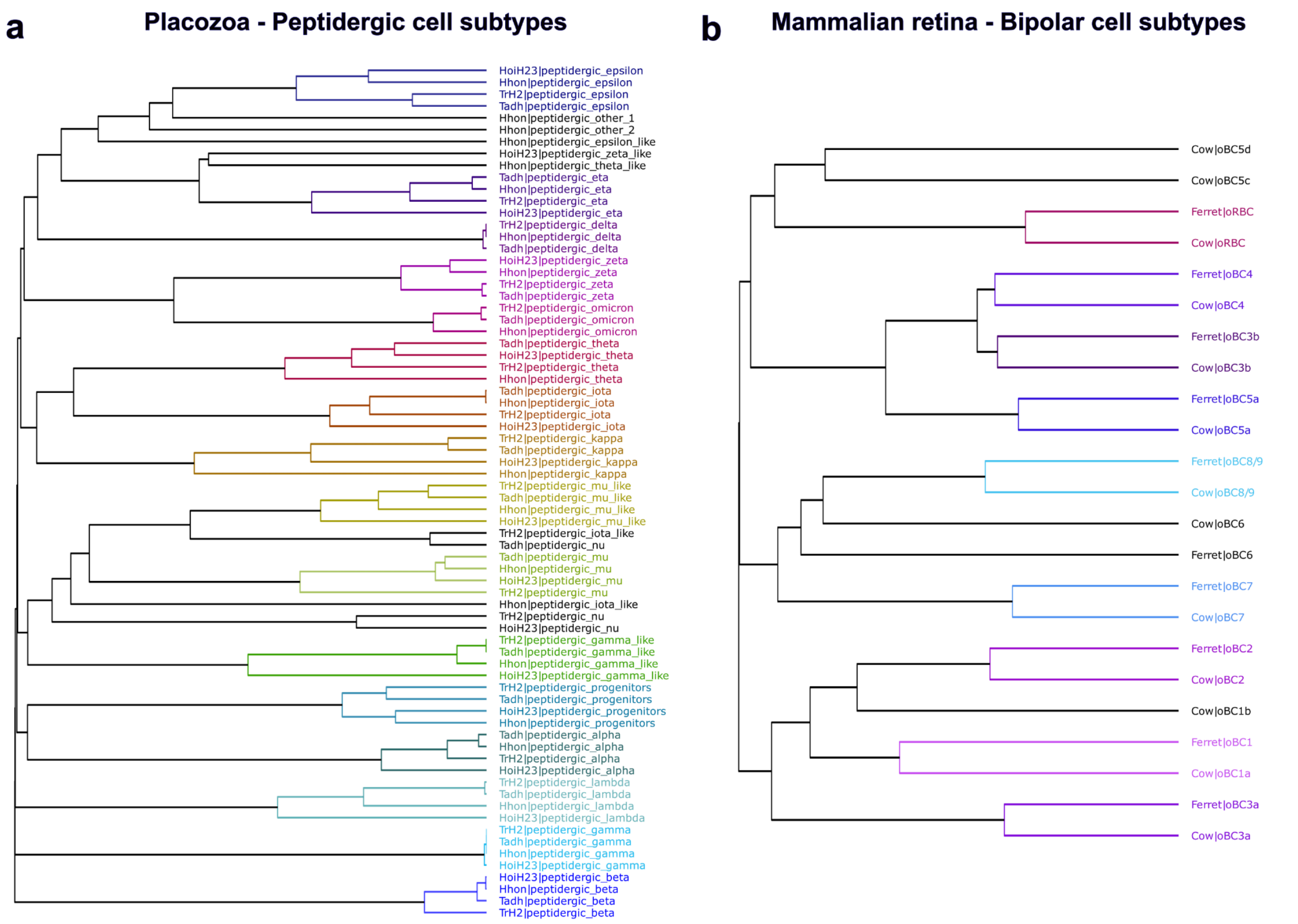

<u>Figure S4:</u> Hierarchical clustering of cell expression profiles for the placozoa whole-body atlas and the mammalian retina dataset. **a.** Hierarchical clustering of placozoan peptidergic cells by Pesci based on all-against-all pairwise comparisons between peptidergic cells only and using --marker_specificity=0.6. Colored groups correspond to cell subtypes annotation from (Najle *et al.*, 2023). Black groups correspond either to (i) species-specific peptidergic cells (epsilon-like, zeta-like and theta-like) (ii) unclassified cells (other_1 and other_2) or (iii) contradictions (iota-like and nu, which are also ambiguous in SAMAp (Najle *et al.*, 2023)). **b.** Hierarchical clustering of Cow and Ferret retinal bipolar cells by Pesci based on all-against-all pairwise comparisons with --marker_specificity=0.6. Colored groups correspond to orthologous subtypes from (Hahn *et al.*, 2023)**.**
